# Retrospective analysis of dietary fat quality and cardiovascular disease risk markers in middle-aged adults

**DOI:** 10.3389/fnut.2025.1699166

**Published:** 2025-11-12

**Authors:** Yongbo Zhang, Fengling Wang, Bingsen Huang, Miaomiao Li, Changchun Du

**Affiliations:** Department of Cardiovascular Medicine, Henan Provincial Chest Hospital, Zhengzhou University, Zhengzhou, China

**Keywords:** fat quality index, cardiovascular biomarkers, hyperlipidemia, inflammation, health

## Abstract

**Background:**

The quality of fat in food affects the cardiovascular system by means of inflammatory and lipid pathways.

**Objective:**

To assess the relationship among middle-aged persons’ cardiovascular disease (CVD) risk indicators and the composition of their dietary fat.

**Methodology:**

Two hundred adults (aged 40–65) from the Henan Provincial Chest Hospital’s databases were incorporated in this retrospective, hospital-based observational investigation. Dietary consumption information was taken from food frequency questionnaires that had been filled out and were kept in medical records. Employing the unsaturated-to-saturated fatty acid proportion (UFA:SFA), dietary fat composition was measured and subjects were separated into tertiles. Anthropometric variables, lipid levels (total cholesterol, LDL-C, HDL-C), and inflammation levels (hs-CRP) were obtained from pre-existing laboratory and hospital records.

**Results:**

In comparison to the lowest tertile, the highest UFA:SFA tertile had greater HDL-C (+8.29 mg/dL, *d* = 1.54), decreased TC (−30.33 mg/dL, *d* = 2.86), LDL-C (−24.73 mg/dL, *d* = 2.67), and hs-CRP (−1.28 mg/L, *d* = 2.53; all *p* < 0.001).

**Conclusion:**

Better lipid and inflammatory levels were associated with higher dietary fat quality, highlighting the significance of substituting unsaturated fats for saturated fats as a CVD prevention measure. However, prospective or interventional research are required to investigate causal effects, as these results are observational and demonstrate association rather than causality.

## 1 Introduction

Dietary fat composition plays a central role in shaping cardiometabolic health. Saturated fatty acids (SFA) are well established to raise low-density lipoprotein cholesterol (LDL-C) and contribute to atherosclerotic cardiovascular disease (CVD), whereas monounsaturated (MUFA) and polyunsaturated fatty acids (PUFA) are generally associated with improved lipid profiles and reduced CVD risk (1). Large-scale evidence, including prospective cohort studies and expert consensus statements, supports replacing SFA with unsaturated fats particularly PUFA to reduce cardiovascular risk at the population level (2, 3). Randomized controlled trials and observational research consistently show reductions in total cholesterol (TC) and LDL-C when SFA are substituted with unsaturated fatty acids, underscoring dietary fat quality as a modifiable determinant of cardiovascular health (4).

Dietary fat quality affects the inflammatory processes that cause atherosclerosis in addition to the typical lipid results. Reduced levels of high-sensitivity C-reactive protein (hs-CRP), a biomarker of systemic inflammation, have been linked to diets high in MUFA and PUFA, such as those that prioritize plant oils, nuts, seeds, and fish (3). Increasingly higher unsaturated-to-saturated fat ratios are associated with stepwise decreases in TC and LDL-C, increases in protective high-density lipoprotein cholesterol (HDL-C), and concurrent decreases in hs-CRP, according to evidence from both population studies and clinical interventions. These results provide a two-pronged strategy for reducing the risk of CVD by highlighting the dual impact of dietary fat quality in altering lipoprotein metabolism and reducing systemic inflammation. It is reported that in improvements in dietary fat quality can reduce hs-CRP independently of weight change, illustrating that the anti-inflammatory benefits of unsaturated fats may be mediated by mechanisms beyond weight reduction (5, 6).

Further evidence for these connections comes from mechanistic understanding. By substituting unsaturated fatty acids for SFA, hepatic lipid synthesis and very-low-density lipoprotein (VLDL) production are modulated, and hepatic LDL receptor expression is increased, enhancing LDL clearance from circulation (7). Toll-like receptor-mediated inflammatory signaling is inhibited and systemic inflammation is reduced as a result of PUFA’s effects on membrane lipid composition and eicosanoid production (4). Increases in HDL-C are both quantitative and functional because consumption of unsaturated fat improves HDL’s ability to efflux cholesterol, as well as its antioxidant and anti-inflammatory qualities (3). These molecular pathways give epidemiologic and clinical data scientific justification, highlighting the significance of dietary fat quality in controlling inflammation and lipids.

Several facets of this association are still poorly described despite a wealth of information. The majority of earlier research has concentrated on clinical or high-risk individuals, while there is relatively little information available on middle-aged, seemingly healthy adults. Additionally, although it offers a more comprehensive picture of metabolic risk, the simultaneous assessment of lipid and inflammatory indicators within the same sample is less frequently reported. By reducing bias from food-based evaluations, the adoption of nutrient-based indices, such as the unsaturated-to-saturated fatty acid ratio, may help enhance comparability. The present retrospective analysis examines the association between dietary fat quality—quantified by the unsaturated-to-saturated fatty acid ratio—and cardiovascular risk markers including TC, LDL-C, HDL-C, and hs-CRP in middle-aged adults without established cardiometabolic disease. This investigation aims to shed more light on the relationship between dietary fat quality and lipid and inflammatory profiles in order to further clarify its function in maintaining cardiovascular health.

## 2 Methodology

### 2.1 Study design and participants

A convenience sample of 200 adults between the ages of 40 and 65 who were chosen from the clinical records of Henan Provincial Chest Hospital between January 2020 and December 2023 was employed in this retrospective research. Individuals had to have their fasting laboratory results and a completed food frequency questionnaire (FFQ) recorded within the same clinical episode in order to be eligible for the investigation. Exclusion criteria included using lipid-lowering or anti-inflammatory medicines, having previous instances of type 2 diabetes, persistent kidney failure, heart illness, or liver condition, or having partial or unavailable dietary records.

### 2.2 Dietary assessment

Information on dietary consumption was taken from Food Frequency Questionnaires (FFQs) that were already filled out and maintained in the documentation (8). The FFQ recorded the typical frequency of consumption and portion sizes of the key food groups and fats throughout the previous 12 months. FFQs were interviewer-administered by qualified dietitians during routine clinical appointments and verified for completeness prior to data entry. Each individual filled out a single, validated semi-quantitative Food Frequency Questionnaire (FFQ), which was administered by the interviewer and incorporated into their medical file, to determine their dietary intake. During the past 12 months, the FFQ inquired about the typical frequency of intake and standard portion size of 110 food items from the main food categories (cereals and grains, vegetables, fruits, meats, fish and shellfish, eggs, dairy, legumes, nuts and seeds, cooking oils, and processed foods). Frequency classifications were “never or <1 time/month” to “≥2 times/day.” Standard household measurements and, if available, portion-size images were used to specify portion sizes. Food consumption information from the FFQ were translated to nutrient and energy intakes by utilizing the food composition database. The consumption of monounsaturated fatty acids (MUFA), polyunsaturated fatty acids (PUFA), and saturated fatty acids (SFA) was measured as a proportion of the overall energy consumed for this study. Unsaturated fatty acid to saturated fatty acid ratio (UFA:SFA) was used to quantify dietary fat quality. This ratio was used to divide individuals into tertiles of dietary fat quality.

### 2.3 Clinical and laboratory measurements

The regularly kept health records of the hospital’s Nutrition and Cardiology departments provided anthropometric, lifestyle, and laboratory information. Laboratory findings were taken from hospital laboratory reports, lifestyle data, including smoking status, was documented in standardized admission or dietary forms, and anthropometric parameters (height, weight, and BMI) were assessed by qualified clinical staff during routine examinations. To reduce temporal disparity between dietary evaluation and biochemical assessments, fasting blood samples and FFQs were taken either within 30 days of one another or during the same clinical appointment. Records that were taken after this time were eliminated.

Each biochemical assessment was taken from standard hospital laboratory records that were gathered during the clinical visits of the patients. Before having their blood drawn, individuals were told to fast for at least 8 h. Following standard operating methods, venous blood was drawn, serum was extracted by centrifugation within 2 h of the draw, and the results were examined at the hospital diagnostic laboratory. A conventional clinical chemistry instrument was used to measure the serum concentrations of total cholesterol (TC), low-density lipoprotein cholesterol (LDL-C), and high-density lipoprotein cholesterol (HDL-C) utilizing automated enzymatic colorimetric techniques. High-sensitivity C-reactive protein (hs-CRP) concentrations were retrieved from immunoturbidimetric tests reported in the same records.

### 2.4 Statistical analysis

The baseline features were presented as frequency (percentages) throughout dietary fat quality tertiles or as averages ± standard deviations (SD). Analysis of covariance (ANCOVA) was used to assess variations in cardiovascular risk indicators after controlling for relevant factors such as age, BMI, gender, total energy intake and smoking status. Covariates were selected *a priori* based on knowledge of their correlation with inflammation and lipid indicators. For pairwise comparisons, Tukey’s *post-hoc* test was used, and *p*-trend estimates were used to evaluate linear trends among tertiles. A *p*-value of less than 0.05 is regarded as statistically significant, and all statistical analyses were carried out using SPSS version 26.0 (IBM Corp., Armonk, NY, United States).

### 2.5 Missing data

Out of the 236 hospital records that were evaluated, 200 patients were incorporated in the final investigation because they contained complete FFQ and laboratory results. Records lacking data on any of the key exposures, outcomes, or covariates were not included.

## 3 Results

### 3.1 Anthropometric and lifestyle characteristics

There was no discernible age-related bias because the average age was similar among the groups. Additionally, the proportions of male and female was comparable, indicating balanced representation of both sexes. The body mass index showed a distinct gradient, reaching its maximum in the lowest tertile and gradually declining in the middle and highest tertiles (*p* < 0.001). This association implies that those who consume more meals high in unsaturated fat typically have lower total body mass. However, BMI should be viewed as indicating variations in total body mass rather than exact body composition, though, because it does not differentiate between fat and lean mass. The incidence of smoking did not follow an even trajectory; the center tertile had somewhat lower proportions than the two extremes (Table 1).

**Table 1:** Baseline characteristics of study participants across tertiles (n = 200).

Variable	T1 (*n* = 67)	T2 (*n* = 66)	T3 (*n* = 67)	*p*-value
Age (years)	52.35 ± 7.97	51.96 ± 6.90	53.86 ± 7.56	0.306
Male, *n* (%)	31 (46.26%)	34 (51.51%)	34 (50.74%)	0.282
BMI (kg/m^2^)	28.53 ± 2.72	27.17 ± 3.40	26.17 ± 3.0	<0.001
Current smoking, *n* (%)	32 (47.76%)	25 (37.87)	31 (46.26%)	0.271
Total energy intake (kcal/day)	2,000 ± 253	2,152 ± 295	2,270 ± 290	<0.001
SFA intake, % energy	14.48	11.10	8.32	<0.001
MUFA intake, % energy	9.22	10.76	13.04	<0.001
PUFA intake, % energy	7.14	8.28	9.49	<0.001
UFA:SFA ratio (mean)	1.15	1.77	2.79	<0.001

Results are presented as mean ± SD, *n* (%), T1: lowest quality, T2: moderate quality, T3: highest quality.

### 3.2 Lipid profile

**Figure 1:**
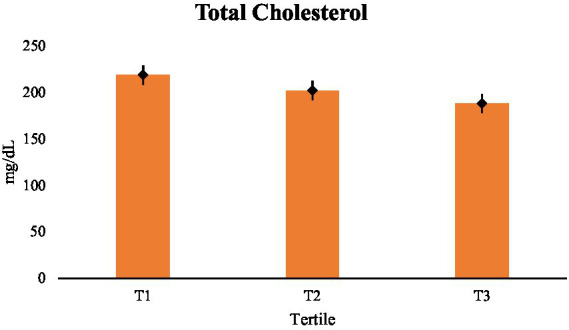
Comparisons of TC (mg/dL) across tertiles.

Table 2 summarizes lipid levels based on tertiles of dietary fat quality. Serum lipid contents and dietary fat quality were shown to be clearly and consistently correlated. Higher dietary fat quality gradually reduced total cholesterol, and all pairwise comparisons were statistically noteworthy (*p* < 0.001). With a distinct dose–response gradient, the *post-hoc* comparisons shown in Table 3 verify that individuals in the highest tertile had substantially reduced cholesterol values compared to individuals in the middle and lowest tertiles (Figure 1).

**Table 2:** Comparison of lipid profile and inflammatory markers.

Variable	T1 (*n* = 67)	T2 (*n* = 66)	T3 (*n* = 67)	*p*-values (unadjusted)	*p*-values (adjusted with BMI)	*p*-values (adjusted without BMI)	Cohen’s *d*	95% CI (adjusted)
Total cholesterol (mg/dL)	218.7 ± 10.7	202.1 ± 10.6	188.0 ± 10.2	<0.001	<0.001	<0.001	2.86	2.28–3.45
LDL-C (mg/dL)	135.6 ± 9.5	122.4 ± 10.5	110.3 ± 7.5	<0.001	<0.001	<0.001	2.67	2.10–3.24
HDL-C (mg/dL)	45.9 ± 5.4	50.7 ± 4.9	54.6 ± 5.7	<0.001	<0.001	<0.001	−1.54	−2.04 to1.04
Hs-CRP (mg/L)	2.8 ± 0.6	2.1 ± 0.5	1.6 ± 0.4	<0.001	<0.001	<0.001	2.53	1.97–3.08

Results are presented as mean ± SD, T1: lowest quality, T2: moderate quality, T3: highest quality.

**Table 3:** *Post-hoc* Tukey comparisons of total cholesterol.

Tertile	Mean difference (with BMI)	Mean difference (without BMI)	*p*_tukey_
T1 vs. T2	16.33	16.27	<0.001
T1 vs. T3	30.33	30.24	<0.001
T2 vs. T3	14.01	13.97	<0.001

The substantial standardized effect sizes (Cohen’s *d* = 2–3) highlight that tertile-based grouping inherently promoted between-group contrast and minimized within-group variance. So, instead of representing causal or interventional impacts, these magnitudes show large cross-sectional separations among extreme dietary-fat-quality categories (Table 2).

**Figure 2:**
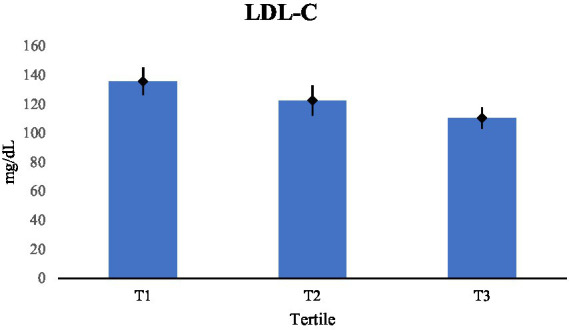
Comparisons of LDL-C (mg/dL) across tertiles.

**Figure 3:**
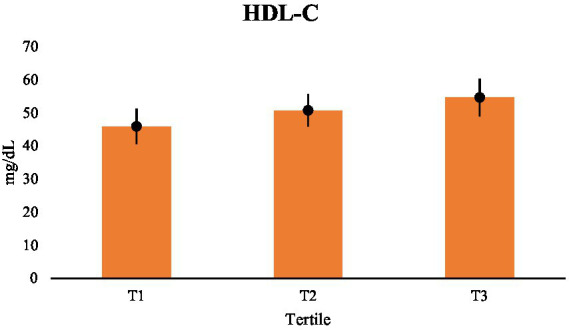
Comparisons of HDL-C (mg/dL) across tertiles.

The same pattern was seen in LDL-C, which decreased across tertiles, with substantial variations shown in Table 4. There was a significant difference between each tertile, suggesting that decreased LDL-C levels were linked to both moderate and major increases in dietary fat quality (Figure 2). Table 2 displays a stepwise boost in HDL-C across tertiles, with Table 5 providing specifics on noteworthy pairwise comparisons. The positive correlation between preventative lipoproteins and fat quality was further supported by the significantly higher HDL-C levels shown by respondents in the top tertile as opposed to the lower tertiles (Figure 3).

**Table 4:** *Post-hoc* Tukey comparisons of LDL-C.

Tertile	Mean difference (with BMI)	Mean difference (without BMI)	*p*_tukey_
T1 vs. T2	12.82	12.72	<0.001
T1 vs. T3	24.73	24.56	<0.001
T2 vs. T3	11.91	11.85	<0.001

**Table 5:** *Post-hoc* Tukey comparisons of HDL-C.

Tertile	Mean difference (with BMI)	Mean difference (without BMI)	*p*_tukey_
T1 vs. T2	−4.55	4.64	<0.001
T1 vs. T3	−8.29	8.44	<0.001
T2 vs. T3	−3.74	3.80	<0.001

Overall, these results show a graded relationship among dietary fat quality and lipid metabolism, wherein higher fat quality is associated with a better lipoprotein profile.

Sensitivity tests with and without BMI adjustments produced similar findings, suggesting that BMI had no bearing on the relationships that were observed. This implies that BMI served more as a confounding factor than a mediating factor (Tables 3–6).

**Table 6:** *Post-hoc* Tukey comparisons of hs-CRP.

Tertile	Mean difference (with BMI)	Mean difference (without BMI)	*p*_tukey_
T1 vs. T2	0.77	0.78	<0.001
T1 vs. T3	1.28	1.29	<0.001
T2 vs. T3	0.50	0.51	<0.001

### 3.3 Inflammation

**Figure 4:**
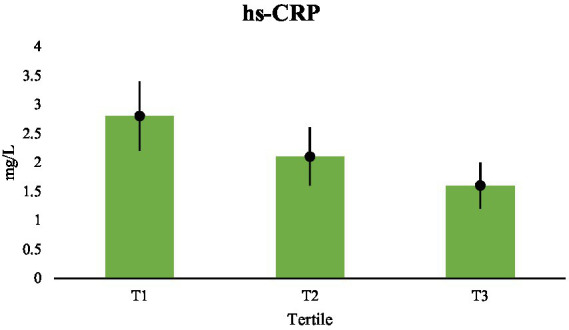
Comparisons of hs-CRP (mg/L) across tertiles.

Additionally, there were substantial differences in high-sensitivity C-reactive protein between tertiles, with decreasing amounts noted as dietary fat quality improved (Table 2). Systemic inflammation was reduced by almost two times when comparing the lowest and highest tertiles. Trend analysis verified a strong linear connection, and all paired comparisons were noteworthy (Table 6). These results show that fat quality has discernible impact on inflammatory processes at the core of cardiovascular disease, extending its effect beyond the metabolism of lipids (Figure 4).

It is notable that the findings are consistent across variables. Higher dietary fat quality was accompanied by concurrent movements in lipid and inflammatory indicators, and the changes were usually profound (*p* < 0.001). The validity of the observed relationships is supported by the uniformity of outcomes across inflammatory and lipid markers; however residual confounding cannot be completely excluded. The scientific plausibility of these relationships is further supported by the graded trend seen over the entire spectrum of dietary fat quality.

The scale of the detected variations has significant cardiovascular risk ramifications. As observed between tertiles, decreases in LDL-C of greater than 20 mg/dL are anticipated to result in a significant drop in long-term cardiovascular events. Likewise, decreases in hs-CRP and increases in HDL-C imply that better fat quality may influence atherosclerosis’s inflammatory and lipid-associated processes. A generally improved cardiometabolic profile linked to the ingestion of unsaturated fat is further supported by the slight decline in BMI. Crucially, these correlations were noted in a group devoid of diabetes, chronic renal or liver diseases, or cardiovascular disease with clinical manifestations. The results thus demonstrate the importance of dietary fat quality as a main preventative strategy.

Model assumption analysis revealed no violations, with homogeneous slopes and no indication of multicollinearity across all factors (Table 7).

**Table 7:** Statistical assumption checks.

Variable	Homogeneity of slopes	Multicollinearity
Total cholesterol	>0.05	VIFs < 1.05
LDL-C	>0.05	VIFs < 1.05
HDL-C	>0.05	VIFs < 1.05
hs-CRP	>0.05	VIF < 1.05

## 4 Discussion

A greater intake of unsaturated-to-saturated fatty acid proportion (UFA:SFA) was linked to reduced levels of total and LDL cholesterol, greater levels of HDL cholesterol, and decreased levels of high-sensitivity C-reactive protein (hs-CRP) in this retrospective investigation of middle-aged adults without a diagnosis of cardiometabolic illness. The design of this research implies these results should only be taken as associations.

### 4.1 Lipid associations

Our outcomes correspond with an extensive body of research showing that a better lipid profile is associated with a larger intake of unsaturated fatty acids. Recent research found that substituting monounsaturated or polyunsaturated fats for saturated fatty acids increased TC:HDL proportions and substantially lowered total and LDL cholesterol (9, 10). Likewise, a meta-analysis demonstrated that diets high in MUFA enhanced lipid indicators without having a negative impact on HDL cholesterol (11). Additional research has shown that consuming more unsaturated fat lowers the probability of cardiovascular disease, which reinforces these observations (1, 12). Moreover, a study reported that substituting plant-based unsaturated fats for saturated fats had comparable benefits on decreasing cholesterol (3). These findings are consistent with their findings. Similarly, a different study confirmed that in free-living people, replacing SFA with MUFA/PUFA results in notable lipid improvements (13). These advantages stem from the fact that unsaturated fats improve HDL particle efficiency and cholesterol transport, while also increasing LDL clearance and decreasing arterial retention (9, 14–16).

### 4.2 Inflammation

One of the main mechanisms connecting food habits and cardiovascular ailments is inflammation (4, 5). After controlling for relevant confounders, the current study demonstrated that blood hs-CRP concentrations were considerably lower in those with higher dietary fat quality. Our study group had a relative decrease of about 44% (from 2.85 to 1.59 mg/L), with the adjusted mean difference between the greatest and lowest fat-quality tertiles being −1.26 mg/L. hs-CRP levels less than 1 mg/L indicate low risk of heart disease, those between 1 and 3 mg/L indicate intermediate danger, and those greater than 3 mg/L indicate serious risk (17). Therefore, even though the average amount in the highest tertile stayed within the intermediate-risk category, the reported shift indicates a significant reduction in systemic inflammation. Increased unsaturated fat consumption may, mechanistically, decrease hs-CRP by inhibiting PPAR-*α* activation and NF-κB signaling, which together diminish cytokine release and hepatic fat storage, thereby moderating systemic inflammation and decreasing IL-6-mediated CRP generation (13, 18–27).

### 4.3 Strengths

This investigation has a number of significant advantages. First, we had the chance to utilize laboratory data that had been assessed using standardized clinical assays by consulting health records, which ensured correctness and reflected actual practice. Second, we were able to record individuals’ typical eating patterns without the effect of research-driven evaluations because the nutritional data was derived from established questionnaires that had previously been filled out as part of regular medical care. Third, the retrospective approach allowed for cost-efficient utilization of previously acquired data to evaluate a sizable sample of middle-aged adults. Lastly, the outcomes are given more legitimacy and biological plausibility by the similar dose–response relationships seen among lipid and inflammatory indicators.

### 4.4 Limitations

It is important to acknowledge the various limitations of this research. Its reliance on material already found in hospital records as a retroactive assessment limited control over the completeness and quality of the material. The study is unable to demonstrate cause-and-effect correlations since eating habits and biomarkers were recorded simultaneously, and reverse causality is still probable. The dietary data was gathered from previous surveys, which may not accurately reflect prolonged habits and are subject to recall bias.

Residual confounding is an inherent limitation of this retrospective analysis. Despite adjustments for measured variables, unaccounted factors such as total diet quality, physical activity, socioeconomic status, and medication use may have influenced the observed associations. Moreover, dietary estimates from food-frequency questionnaires are prone to recall and reporting error, underscoring the need for prospective or interventional studies to clarify causal pathways.

In addition, the external validity of the results is restricted by the convenience-based data collection from medical records. Individuals who seek medical advice or have biochemical testing done may exhibit systemic differences in subclinical illness burden, lifestyle, or health literacy compared to the general population. Consequently, although the research offers valuable understanding of correlations within an actual clinical group, the findings might not be immediately applicable to larger community-based groups. It is recommended that future research employ multi-center recruitment or population-based random sampling to improve representation and validate these correlations in a variety of demographic and lifestyle scenarios.

### 4.5 Future recommendation

In order to establish causal links between dietary fat quality, lipid metabolism, and systemic inflammation, future research should build on these findings by using randomized controlled trials and longitudinal cohort studies. Mechanistic research is also necessary to investigate the molecular effects of diets high in MUFAs and PUFAs on lipoprotein function, cholesterol transport, and inflammatory signaling pathways. External validity would be enhanced and conclusions would have wider relevance if studies were extended to a wider range of populations with different genetic and lifestyle backgrounds. These results highlight the value of encouraging small changes in dietary fat composition, such as substituting MUFA and PUFA for saturated fats, as a scalable and affordable strategy to lower the risk of cardiovascular disease from a public health standpoint. Even bigger gains in cardiometabolic health outcomes may result from combining dietary changes with complementary lifestyle practices, such as encouraging physical activity, quitting smoking, and managing weight.

## 5 Conclusion

This study showed that in middle-aged adults without a history of cardiometabolic disease, better dietary fat quality was associated with favorable lipid and inflammatory profiles. Along with greater HDL-C, participants in the highest tertile showed significantly reduced levels of total cholesterol, LDL-C, and hs-CRP, indicating both lipid-lowering and anti-inflammatory advantages. These connections’ biological plausibility is strengthened by the consistent dose–response gradients. Crucially, the results imply that even small increases in the quality of dietary fat could have a significant impact on lowering the risk of heart disease. In sum, encouraging diets high in MUFA and PUFA is an economical method that may have a significant impact on cardiac health.

## Data Availability

The raw data supporting the conclusions of this article will be made available by the authors, without undue reservation.
